# Wide versus narrow excision margins for high-risk, primary cutaneous melanomas: long-term follow-up of survival in a randomised trial

**DOI:** 10.1016/S1470-2045(15)00482-9

**Published:** 2016-02

**Authors:** Andrew J Hayes, Lauren Maynard, Gillian Coombes, Julia Newton-Bishop, Michael Timmons, Martin Cook, Jeffrey Theaker, Judith M Bliss, J Meirion Thomas

**Affiliations:** a The Royal Marsden Hospital National Health Foundation Trust, London, UK; b Clinical Trials and Statistics Unit at The Institute of Cancer Research, London, UK; c Leeds Institute of Cancer and Pathology, Leeds, UK; d Bradford Royal Infirmary, Bradford, UK; e Royal Surrey County Hospital, Guildford, UK; f University Hospitals Southampton, Southampton, UK

## Abstract

**Background:**

The necessary margin of excision for cutaneous melanomas greater than 2 mm in thickness is controversial. At a median follow-up of 5 years, findings from our previously published randomised trial of narrow (1 cm) versus wide (3 cm) excision margins in patients with thick cutaneous melanomas showed that narrow margins were associated with an increased frequency of locoregional relapse, but no significant difference in overall survival was apparent. We now report a long-term survival analysis of that trial.

**Methods:**

We did a randomised, open-label multicentre trial in 59 hospitals—57 in the UK, one in Poland, and one in South Africa. Patients with one primary localised cutaneous melanoma greater than 2 mm in Breslow thickness on the trunk or limbs (excluding palms or soles) were randomly assigned (1:1) centrally to receive surgery with either a 1 cm or 3 cm excision margin following an initial surgery. The randomisation lists were generated with random permuted blocks and stratified by centre and extent of initial surgery. The endpoints of this analysis were overall survival and melanoma-specific survival. Analyses were done in the intention-to-treat population. This trial was not registered because it predated mandatory trial registration.

**Findings:**

Between Dec 16, 1992, and May 22, 2001, we randomly assigned 900 patients to surgery with either a 1 cm excision margin (n=453) or a 3 cm excision margin (n=447). At a median follow-up of 8·8 years (106 months [IQR 76–135], 494 patients had died, with 359 of these deaths attributed to melanoma. 194 deaths were attributed to melanoma in the 1 cm group compared with 165 in the 3 cm group (unadjusted hazard ratio [HR] 1·24 [95% CI 1·01–1·53]; p=0·041). Although a higher number of deaths overall occurred in the 1 cm group compared with the 3 cm group (253 *vs* 241), the difference was not significant (unadjusted HR 1·14 [95% CI 0·96–1·36]; p=0·14). Surgical complications were reported in 35 (8%) patients in the 1 cm excision margin group and 65 (15%) patients in the 3 cm group.

**Interpretation:**

Our findings suggest that a 1 cm excision margin is inadequate for cutaneous melanoma with Breslow thickness greater than 2 mm on the trunk and limbs. Current guidelines advise a 2 cm margin for melanomas greater than 2 mm in thickness but only a 1 cm margin for thinner melanomas. The adequacy of a 1 cm margin for thinner melanomas with poor prognostic features should be addressed in future randomised studies.

**Funding:**

Cancer Research UK, North Thames National Health Service Executive, Northern and Yorkshire National Health Service Executive, British United Provident Association Foundation, British Association of Plastic Surgeons, the Meirion Thomas Cancer Research Fund, and the National Institute for Health and Research Biomedical Research Centre at The Royal Marsden NHS Foundation Trust.

## Introduction

The risk of metastatic spread from a malignant melanoma is estimated on the basis of histopathological features such as the Breslow thickness, mitotic rate, and the presence of microscopic ulceration.^1^, ^2^ Whether the surgical margins that are taken around the primary tumour affect metastatic spread is unclear, despite having been the subject of several randomised clinical trials.^3^, ^4^, ^5^, ^6^, ^7^ Historically, wide surgical margins of 5 cm or more were taken around primary melanomas in an attempt to not only excise the primary tumour but also to encompass local micrometastatic disease in the vicinity of the tumour.^8^, ^9^ In findings from all previously reported randomised trials comparing wide (3–5 cm) with narrow (1–2 cm) margins, no significant difference in overall survival between the groups has been reported. For melanoma-specific survival, although no trial has shown a significant differential risk associated with different surgical margin widths, findings from two trials^3^, ^4^ and the previous report of this trial^10^ suggest a possible detrimental effect of narrow margins on melanoma-specific survival. In a Swedish Melanoma Study Group trial^3^ of patients randomly assigned to excision margins of either 2 cm or 5 cm for trunk and extremity melanomas with a Breslow thickness 0·8–2·0 mm (median 1·2 mm), the hazard ratio (HR) for melanoma deaths for narrow margins compared with wide margins was 1·22 (95% CI 0·88–1·69; p=0·24) with a median follow-up of 11 years. Additionally, the Intergroup Melanoma Surgical Trial^4^ of patients randomly assigned to either a 2 cm or 4 cm excision margin for trunk and extremity melanomas with Breslow thickness 1–4 mm (median 1·96 mm) showed a non-significant difference (p=0·07) in 10-year disease-specific survival between groups (70% for the 2 cm group and 77% for the 4 cm group). However, findings from a second Swedish Melanoma Study Group trial^6^ that randomly assigned patients with melanomas of Breslow thickness greater than 2 mm (median 3·1 mm) to either a 2 cm or a 4 cm excision margin showed no difference in melanoma-specific survival between the two groups (HR 0·99 [95% CI 0·78–1·26]; p=0·95).

**Research in context**

**Evidence before this study**

We searched PubMed for articles published up to Aug 1, 2015, on randomised trials assessing surgical excision margins in cutaneous melanoma, using the search terms “melanoma”, “margins”, “excision”, and “surgery”. No languages were excluded from the search. We identified five randomised trials that analysed the effect of surgical margins on outcomes in cutaneous melanoma. None of these trials have shown a significant effect of the choice of surgical margins on either locoregional relapse or melanoma-specific survival. However, some of these studies have shown non-significant results favouring wider margins in minimising locoregional and distant relapse.

**Added value of this study**

This study reports on long-term survival analysis and, to our knowledge, is the first to show that wider surgical margins result in a significant improvement in melanoma-specific survival.

**Implications of all the available evidence**

This study, alongside the other randomised trials, reiterates current international guidelines stating that a 1 cm margin is inadequate for the treatment of a melanoma greater than 2 mm in Breslow thickness. It lends support to further investigation of the adequacy of a 1 cm margin for melanomas between 1 mm and 2 mm in thickness, especially those with other poor prognostic features, for which most international guidelines at present still advise a 1 cm excision.

In 2004, we reported the findings from our randomised clinical trial^10^ of patients with high-risk malignant melanoma (Breslow thickness ≥2 mm) randomly assigned to excision margins of either 1 cm or 3 cm, with a median follow-up of 5 years. We showed a negative association between narrow margins and locoregional relapse-free survival (defined as local recurrence, in-transit metastases, and regional lymph node metastases); ie, a 1 cm margin was associated with a significantly greater risk of locoregional recurrence than the 3 cm margin (multivariable adjusted HR 1·34 [95% CI 1·06–1·71]; p=0·02). No significant differences were apparent between the two groups for either melanoma-specific survival or overall survival. We now report an extended follow-up of the survival endpoints of this trial with a median follow-up of 8·8 years.

## Methods

### Study design and participants

We have previously described the study design, patient eligibility criteria, trial protocol, and endpoints of the randomised trial in detail.^10^ Briefly, between Dec 16, 1992, and May 22, 2001, we did a randomised, open-label multicentre trial in 59 centres in the UK, South Africa, and Poland. The trial was done under the auspices of the UK Melanoma Study Group, the British Association of Plastic Surgeons, and the Scottish Cancer Therapy Network and was approved by the local ethics committees of all participating centres. Patients aged 18 years or older with one primary localised cutaneous melanoma greater than 2 mm in Breslow thickness arising on the trunk or limbs (but not including the soles of feet or palms of hands) were eligible for the study. Before randomisation, the primary melanoma was excised in an initial surgery with either a 1 mm (proposed pathway) or 1 cm (alternative pathway) margin of excision. A diagnosis of melanoma with a Breslow thickness greater than 2 mm was confirmed histopathologically by histopathologists in participating centres. Two histopathologists from the trial management group did a histopathological review of the primary melanoma in all cases. Patients could not have any previous history of cancer, other than basal cell carcinoma. Written informed consent was obtained for all participants. This trial was not registered as it predated mandatory trial registration.

### Randomisation and masking

We randomly assigned patients (1:1) to either a 1 cm surgical excision or a 3 cm surgical excision as the measured clinical margin taken around the primary melanoma lesion. We did the randomisation centrally by telephone call from the patient's treating centre to The Institute of Cancer Research Clinical Trials and Statistics Unit (ICR-CTSU). Randomisation lists were generated at the ICR-CTSU with random permuted blocks and stratified according to centre and extent of initial surgery. Treatment allocation was not masked.

### Procedures

Patients who had a 1 mm initial excision (proposed pathway) received either a further 1 cm or 3 cm excision. Patients who had a 1 cm initial excision (alternative pathway) received either no further treatment or a further 2 cm excision. If further surgery was needed after the initial excision, it had to be done within 45 days. The method of surgical closure was at the discretion of the participating surgeon. Elective lymph node dissection and sentinel node biopsy were not part of routine practice at the time the trial was undertaken and adjuvant chemotherapy was not allowed in the trial protocol. After treatment, patients were followed up for local and distant relapse and death every 3 months for the first 2 years, then every 6 months for up to 5 years, and annually thereafter. Assessment of disease at the point of relapse was by standard clinical practice within participating centres at that time. Data from trial case report forms (when available) were used to identify cause of death. To maximise the amount of survival data obtained, we also traced UK patients (n=790) for their vital status and in January, 2012, we requested the death certificates of patients known to have died to identify the cause of death as stated on the certificate. Death certificates were not obtained for non-UK patients (n=110). Death was classed as melanoma-specific if the cause of death was reported as melanoma on the clinical trial case-report form for death, or if any evidence was present of distant metastatic melanoma at the time of death (as reported in patient files or on death certificates). Attribution of the cause of death as stated on the death certificate was masked to clinical records and treatment group.

### Outcomes

The primary endpoints of the original trial^10^ were locoregional recurrence and disease-free survival. Because of our restricted ability to obtain recurrence data in later years, these endpoints were not reassessed in the present long-term analysis. Instead, we assessed the original secondary endpoints of overall survival, measured as time from randomisation to death from any cause, and melanoma-specific survival, measured as time from randomisation to death reported to be from melanoma. Data for surgical complications were obtained as part of the original analysis.

### Statistical analysis

The original planned sample size was 600 patients, based on an expected 3-year local or in-transit recurrence rate of 15%; however, because of a lower recurrence rate than expected, the sample size was increased to 900 after discussion with the trial management group and the data monitoring committee.^10^ At the time of writing the protocol, we expected that 40–50% of patients with a 3 cm excision would have disease recurrence within 2–3 years. At the time patients were recruited, survival after diagnosis of metastatic relapse was understood to be poor. The protocol did not specify a sample size for the secondary survival endpoints. Unless otherwise stated, we did all analyses in the intention-to-treat population, which includes all randomly assigned patients. For overall survival, patients not known to have died were censored at the date of last follow-up. For melanoma-specific survival, patients who died of non-melanoma causes were censored at the time of death and patients who died from an unknown cause were censored on the day before their date of death. Both UK and non-UK patients who were not known to have died were censored at the date of their last visit. To assess the robustness of these assumptions we did a competing-risks analysis, treating confirmed non-melanoma deaths as the competing event.

We constructed Kaplan-Meier curves^11^ and calculated HRs using the Cox proportional hazards model;^12^ we compared treatment groups using the log-rank test. We calculated absolute risk difference at 10 years with normal estimated 95% CIs and assessed the effect of individual prognostic factors in a multivariable analysis using the same Cox proportional hazards model as in our previous report,^10^ adjusting for age. Variables are included in the model as categorical indicators, other than tumour thickness which was classified as 0–2·49 mm, 2·50–3·49 mm, 3·50–4·49 mm, 4·50–5·49 mm, and 5·50 mm or more, and fitted as a linear effect. HRs (with 95% CIs) are presented, with HRs greater than 1 suggesting a disadvantage to the 1 cm margin group relative to the 3 cm margin group. We tested the proportionality assumption of the Cox model with Schoenfeld residuals. For the competing risks analysis, we plotted cumulative incidence functions for each cause of death (melanoma and non-melanoma) and compared treatment groups with Gray's test. HRs were obtained from the univariate Fine and Gray model.^13^, ^14^ We also did a multivariable analysis with the Fine and Gray model, including the same variables as the multivariable Cox model. To explore the effect of age on cause of death, patients were classified at randomisation in five age groups containing about equal numbers of patients (<45 years, 45–53 years, 54–63 years, 64–70 years, and ≥71 years), defined using quintiles and rounding to the nearest whole number. We estimated the rates of death from melanoma and other causes for each age group as the probability of dying from a specific cause in 2-year intervals from randomisation if the patient was alive at the beginning of each 2-year interval, using cumulative incidence functions from the competing risks analysis.

Because we obtained death certificates for UK patients only, we did a sensitivity analysis including UK patients only. For this analysis we constructed Kaplan-Meier curves and calculated HRs using the Cox proportional hazards model for UK patients only.

We did a post-hoc subgroup analysis to assess whether sex, tumour thickness, age group, site, ulceration, and proposed versus alternative pathway initial excision were associated with treatment effect. We used Wald tests to compare the HRs between subgroups. To compensate for multiplicity for subgroup analyses, p values of less than 0·01 were deemed significant, therefore these HRs are presented with corresponding 99% CIs. We used two-sided significance tests throughout.

This was the first and only analysis that had been done on this dataset since the initial report of this trial. We did the competing risks analysis with R 3.0.2 and all other analyses with Stata version 11.2.

### Role of the funding source

The funders of the study had no role in study design, data collection, data analysis, data interpretation, or writing of the report. The corresponding author had full access to all the data in the study and had final responsibility for the decision to submit for publication.

## Results

**Figure 1:**
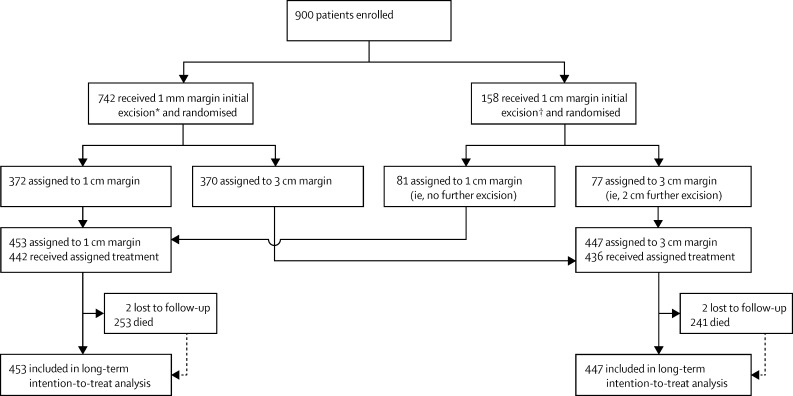
Trial profile *Initial excision by the proposed pathway. †Initial excision by the alternative pathway.

Between Dec 16, 1992, and May 22, 2001, 900 patients were enrolled at 57 hospitals in the UK, one hospital in Poland, and one hospital in South Africa (appendix) and received an initial excision of 1 mm or 1 cm, and were then randomly allocated to either a total 1 cm surgical margin (n=453) or a total 3 cm surgical margin (n=447; figure 1). 442 (98%) patients in the 1 cm group and 436 (98%) patients in the 3 cm group underwent the allocated treatment. Baseline characteristics were well balanced between the two groups (table 1). Median follow-up for all patients was 5·7 years (68 months [IQR 35–103]) and median follow-up in patients not known to have died (censoring at death) was 8·8 years (106 months [76–135]). Median follow-up for the UK patients for whom a death certificate analysis was done (censoring at death) was 9·3 years (111 months [IQR 82–141]) and median follow-up in non-UK patients was 6·3 years (76 months [62–100]). 494 deaths were reported; 359 of these were death from melanoma. For three participants (two in the 1 cm margin group, one in the 3 cm group), melanoma was present at the time of death but this was not the cause of death. Cause of death was unknown for ten patients (three in the 1 cm margin group, seven in the 3 cm group). Four patients did not have any follow-up after randomisation and were censored at randomisation.

**Figure 2:**
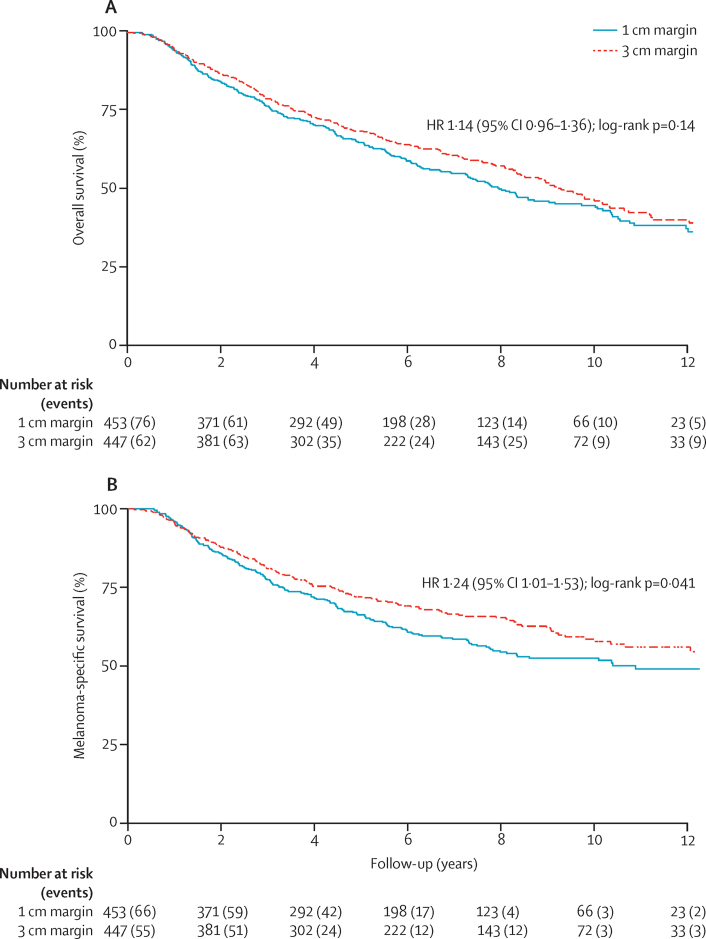
Overall survival (A) and melanoma-specific survival (B) HR=hazard ratio.

The snapshot used for the current analysis was taken on Aug 31, 2012, after information from the death certificate analysis had been obtained. 253 deaths occurred overall in 453 patients in the 1 cm margin group and 241 occurred in 447 patients in the 3 cm group (unadjusted HR 1·14 [95% CI 0·96–1·36]; p=0·14; figure 2). 194 deaths attributed to melanoma occurred in the 1 cm margin group compared with 165 in the 3 cm margin group (unadjusted HR 1·24 [95% CI 1·01–1·53]; p=0·041; figure 2). The estimated absolute difference in melanoma-specific survival at 10 years between the two groups was 5·95% (95% CI −0·54 to 12·44). The proportionality assumption of the Cox model was not violated (appendix). Results from the sensitivity analysis of UK patients only (n=338 in the 1 cm margin group; n=392 in the 3 cm margin group) showed HRs of 1·11 (95% CI 0·92–1·33) for overall survival and 1·21 (0·97–1·50) for melanoma-specific survival; the results were consistent with the primary result.

**Figure 3:**
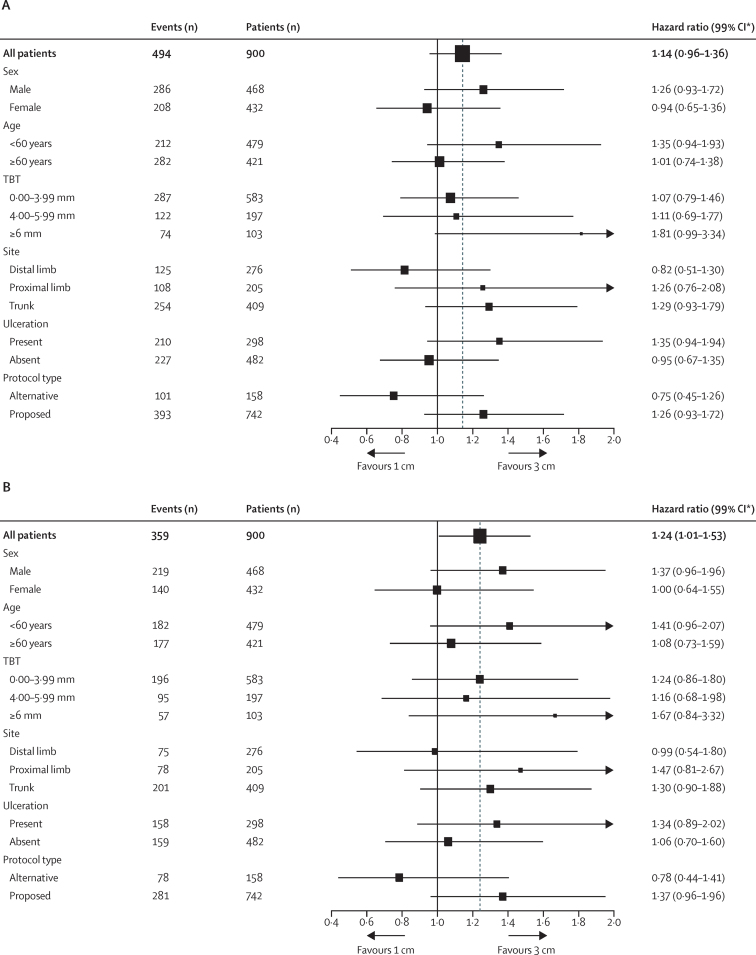
Univariable subgroup analyses of overall survival (A) and melanoma-specific survival (B) The dotted line shows the hazard ratio for all patients. Data excludes patients with unknown values for each variable. TBT=total Breslow thickness. *95% CIs presented for all patients; 99% CIs presented for subgroups.

773 patients (385 in the 1 cm group and 388 in the 3 cm group) had complete data for all known prognostic factors and were included in a multivariable analysis (table 2). Having adjusted for known prognostic factors, the effect of a 1 cm margin versus a 3 cm margin was similar to that found in the unadjusted analysis. When we assessed interactions between margin width and sex, tumour thickness, age group, site, and ulceration in a subgroup analysis, none were significant for either overall survival or melanoma-specific survival (figure 3).

**Figure 4:**
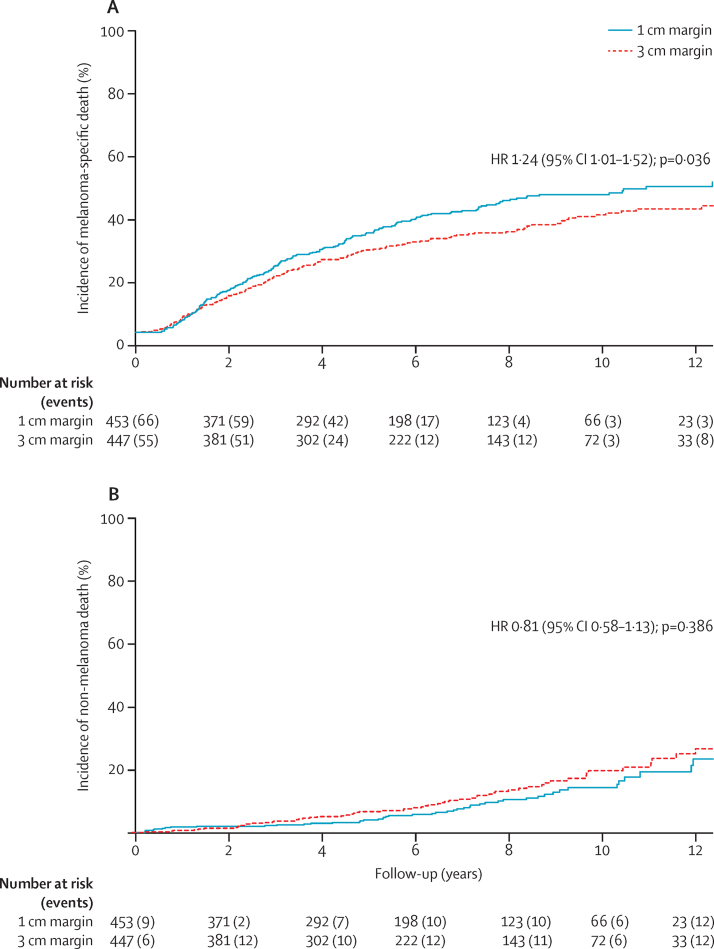
Cumulative incidence for death due to melanoma (A) and death due to other causes (B) HR=hazard ratio.

When the effect of competing deaths due to other causes was taken in account, the cumulative incidence of death from melanoma was higher in the 1 cm margin group than in the 3 cm margin group (figure 4; point estimates of cumulative incidence at 8·8 years were 47·9% [95% CI 42·8–53·2] in the 1 cm margin group, 38·1% [33·3–43·4] in the 3 cm group). The multivariable analysis done with Fine and Gray's model for melanoma-specific deaths showed similar results to the Cox model for melanoma-specific survival (appendix). No differences between the two margin widths were noted regarding the cumulative incidence of death due to other causes (figure 4; point estimates of cumulative incidence at 8·8 years were 14·5% [95% CI 10·1–20·6] in the 1 cm margin group, 18·2% [13·6–24·0] in the 3 cm group). The association of prognostic factors with non-melanoma death was assessed; only age was found to be associated (appendix). Deaths from melanoma and non-melanoma causes over time in all age groups are shown in table 3. The number of non-melanoma deaths in younger age groups was negligible, but became more predominant in later years of follow-up for older patients (table 3). Surgical complications were reported in 35 (8%) of 453 patients in the 1 cm group and 65 (15%) of 447 patients in the 3 cm group. The most common complications were partial or complete graft loss (ten [2%] in the 1 cm group, 20 [4%] in the 3 cm group) and wound dehiscence (seven [2%] in the 1 cm group, nine [2%] in the 3 cm group).

## Discussion

At a median follow-up of 8·8 years (106 months) the risk of death from melanoma was significantly higher in the narrow (1 cm) margin group than in the wider (3 cm) margin group. The poorer prognosis from melanoma associated with the 1 cm margin of excision was not specific to any particular subgroup of patients. The estimated risk of death from any cause was higher in the 1 cm margin group than in the 3 cm margin group, although this difference was not significant. A limitation of our study is that complete data were not available for all patients; in particular, death certificates were not obtained for non-UK patients. However, the results of a sensitivity analysis of UK patients only were consistent with the primary results.

Findings from our previous report of this trial^10^ with a median follow-up of 5 years showed that, in patients with high-risk melanoma, a 1 cm excision margin was associated with a significant increase in locoregional relapse compared with a 3 cm excision margin. In that report, although the number of deaths differed between the two study groups, the difference was not significant (128 deaths from melanoma in the group with 1 cm excision margins compared with 105 in the group with 3 cm excision margins; HR 1·24 [95% CI 0·96–1·61], p=0·1). Additionally, no differences were noted in overall survival between the two groups (32·2% in the 1 cm group *vs* 30·9% in the 3 cm group; HR 1·07 [95% CI 0·75–1·36], p=0·6).

The current analysis does not include an updated analysis of the locoregional recurrence endpoint because follow-up data for locoregional relapse beyond 5 years are sparse. This report describes analyses only of melanoma-specific survival and overall survival, and shows that a narrow margin significantly reduced melanoma-specific survival compared with a wider margin. Locoregional relapse is the most common first site of relapse of metastatic melanoma and, accordingly, an increased risk of locoregional relapse in the narrow margin group might suggest an increased future risk of melanoma-specific death. During the period of this study, when no effective systemic therapies for metastatic melanoma were available, stage IV disease was associated with a very poor prognosis with a median survival of between 8 and 18 months depending on the pattern of metastatic spread.^15^ Although age, sex, tumour thickness, ulceration, and tumour site all seem to be prognostic factors for overall survival, only age was shown to affect non-melanoma deaths, which occurred in similar numbers in the 1 cm and the 3 cm groups.

Because sentinel node biopsy was not done routinely in this trial,^10^ the trial groups might have been imbalanced in terms of clinically occult disease within regional lymph nodes at the time of randomisation, which could have biased the outcome of the trial. Also the prognostic importance of mitotic rate, lymphovascular invasion, and microsatellitosis was not known when this study was initiated. However, treatment allocation was by randomisation, which aims to ensure no systematic differences exist between the two groups for both known and unknown prognostic factors. Any imbalances in unobserved factors in this study would be due to chance and a chance imbalance in a study of this size is unlikely to affect the outcomes. Because the trial groups were well balanced in terms of other known prognostic factors for outcome at the time of trial recruitment (eg, sex, tumour thickness, disease site) and ulceration was slightly more prevalent in the 3 cm group, the sentinel node status is unlikely to be worse in the 1 cm group than in the 3 cm group, although a chance imbalance remains possible.

This study cannot establish whether locoregional relapse predisposes patients to the subsequent development of distant metastatic disease, or whether the development of locoregional disease is merely correlated with and predates the development of metastatic disease. However, a 1 cm clinical margin should be adequate to completely excise a primary melanoma with negative microscopic margins. Because a 3 cm margin resulted in a decreased number of melanoma deaths, this would suggest that surgically intervening in a micrometastatic process in the 3 cm around the primary tumour can somehow affect the later metastatic process at more distant sites. Previous studies have shown a significant increase in local recurrence rates after 1 cm excisions,^6^, ^16^ suggesting that 1 cm margins might not be adequate to deal with local microsatellitosis.

Previous randomised studies of elective^17^, ^18^, ^19^, ^20^, ^21^ or selective^22^ lymph node dissection have not shown a significant difference in melanoma-specific survival. The absence of a proven survival benefit in these nodal studies is at odds with the probable biological hypothesis for an effect on survival shown in this study—ie, that removal of microsatellites around the primary tumour affects the development of metastatic disease. However, because subgroup analyses in these studies raised the possibility of survival benefit for prophylactic lymph node clearance that was not detected at the point of randomisation,^22^ there might be a consistent biological process underlying the effect noted in this study and in previous studies of prophylactic lymph node clearance.

Current international guidelines advise a 2 cm excision for melanomas greater than 2 mm in thickness and findings from the other major randomised study for thick melanomas^6^ suggested that a 4 cm excision was not better than a 2 cm excision in terms of melanoma-specific survival. Hence, although the findings from our study suggest that a 1 cm margin seems inadequate for excision of melanomas thicker than 2 mm, margins greater than 2 cm need not necessarily be taken. Our study has re-emphasised that the choice of surgical margins taken around a cutaneous melanoma is important and, to our knowledge, for the first time provides evidence to suggest that a narrower excision margin used for thick primary tumours affects melanoma-specific survival. This finding might be pertinent for specific melanomas for which narrow (1 cm) margins are presently advised—ie, melanomas between 1 mm and 2 mm in thickness with other adverse prognostic features (ulceration or high mitotic rate, or both). The possible difference between a 1 cm and 2 cm margin for melanomas greater than 1 mm in thickness is being investigated in a randomised trial in progress (NCT02385214).

## Figures and Tables

**Table 1:** Baseline characteristics of the intention-to-treat population

		**1 cm excision margin (n=453)**	**3 cm excision margin (n=447)**
Sex			
	Male	248 (55%)	220 (49%)
	Female	205 (45%)	227 (51%)
Age (years)			
	<60	243 (54%)	236 (53%)
	≥60	210 (46%)	211 (47%)
	Median	58·7 (47·1–68·8)	58·7 (47·3–70·1)
Tumour thickness (mm)			
	<2·50	133 (29%)	114 (26%)
	2·50–3·49	136 (30%)	144 (32%)
	3·50–4·49	77 (17%)	77 (17%)
	4·50–5·49	40 (9%)	40 (9%)
	≥5·50	65 (14%)	72 (16%)
	Missing information	2 (<1%)	0
	Median	3 (2·3–4·2)	3·1 (2·4–4·5)
Tumour thickness (mm)*			
	≤1·00	0	2 (<1%)
	1·01–2·00	55 (12%)	44 (10%)
	2·01–4·00	280 (62%)	275 (62%)
	>4·00	116 (26%)	126 (28%)
	Missing information	2 (<1%)	0
Site			
	Distal	136 (30%)	140 (31%)
	Proximal	108 (24%)	97 (22%)
	Trunk	203 (45%)	206 (46%)
	Missing information	6 (1%)	4 (<1%)
Ulceration (>1 mm)			
	Absent	249 (55%)	233 (52%)
	Present	144 (32%)	154 (35%)
	Not assessed	60 (13%)	60 (13%)
Initial surgery			
	Proposed (1 mm)	372 (82%)	370 (83%)
	Alternative (1 cm)	81 (18%)	77 (17%)

Data are n (%) or median (IQR).

* UICC classification.

**Table 2:** Multivariable analysis of prognostic factors in overall survival and melanoma-specific survival

		**n (%)**	**Overall survival**	**Melanoma-specific survival**
Excision margin				
	3 cm	388 (50%)	1·00*	1·00*
	1 cm	385 (50%)	1·19 (0·99–1·45)	1·28 (1·02–1·61)
	p value	..	0·070	0·031
Sex				
	Female	354 (46%)	1·00*	1·00*
	Male	419 (54%)	1·38 (1·11–1·71)	1·38 (1·07–1·77)
	p value	..	0·0035	0·013
Tumour thickness† (mm)		773 (100%)	1·18 (1·10–1·27)	1·23 (1·13–1·33)
	p value	..	<0·0001	<0·0001
Ulceration				
	Absent	477 (62%)	1·00*	1·00*
	Present	296 (38%)	1·68 (1·38–2·04)	1·74 (1·39–2·19)
	p value	..	<0·0001	<0·0001
Site				
	Distal limb	244 (32%)	1·00*	1·00*
	Proximal limb	174 (23%)	1·23 (0·93–1·63)	1·46 (1·04–2·05)
	Trunk	355 (46%)	1·41 (1·09–1·81)	1·71 (1·26–2·31)
	p value	..	0·029	0·0026
Age (years)				
	<60	400 (52%)	1·00*	1·00*
	≥60	373 (48%)	1·49 (1·23–1·81)	1·12 (0·89–1·39)
	p value	..	0·0001	0·34

Data are n (%), hazard ratio (95% CI) of 773 patients with available data. p values from Wald test.

* Reference group.

† Tumour thicknesses classified as 0·00–2·49 mm, 2·50–3·49 mm, 3·50–4·49 mm, 4·50–5·49 mm, and ≥5·50 mm, and fitted as linear effects.

**Table 3:** Deaths from melanoma and other causes in different age groups

		**<45 years (n=180)**	**45–53 years (n=185)**	**54–63 years (n=190)**	**64–70 years (n=161)**	**≥71 years (n=184)**
Patients alive at 2 years		159 (88%)	157 (85%)	155 (82%)	143 (89%)	138 (75%)
Total deaths ≤2 years		18	27	30	15	46
	Deaths from melanoma	17 (11%)	27 (17%)	27 (17%)	14 (10%)	36 (26%)
	Deaths from other cause(s)	1 (<1%)	0	3 (2%)	1 (<1%)	10 (7%)
Patients alive at 4 years		121 (67%)	129 (70%)	127 (67%)	112 (70%)	105 (57%)
Total deaths from 2·01–4·00 years		29	22	17	24	32
	Deaths from melanoma	29 (23%)	21 (16%)	17 (13%)	22 (19%)	21 (20%)
	Deaths from other cause(s)	0	1 (<1%)	0	2 (2%)	11 (11%)
Patients alive at 6 years		80 (44%)	90 (49%)	93 (49%)	76 (47%)	81 (44%)
Total deaths from 4·01–6·00 years		13	13	16	21	20
	Deaths from melanoma	13 (14%)	12 (11%)	13 (13%)	16 (20%)	12 (15%)
	Deaths from other cause(s)	0	1 (1%)	3 (3%)	5 (6%)	8 (10%)
Patients alive at 8 years		49 (27%)	60 (32%)	56 (29%)	47 (29%)	54 (29%)
Total deaths from 6·01–8·00 years		5	4	12	14	16
	Deaths from melanoma	4 (7%)	2 (3%)	7 (11%)	10 (19%)	6 (10%)
	Deaths from other cause(s)	1 (2%)	2 (3%)	5 (7%)	4 (7%)	10 (17%)
Patients alive at 10 years		30 (17%)	29 (16%)	30 (16%)	26 (16%)	23 (13%)
Total deaths from 8·01–10·00 years		5	5	1	5	21
	Deaths from melanoma	5 (14%)	3 (7%)	0	1 (3%)	7 (24%)
	Deaths from other cause(s)	0	2 (4%)	1 (3%)	4 (12%)	14 (50%)
Patients alive at 12 years		8 (4%)	11 (6%)	15 (8%)	9 (6%)	13 (7%)
Total deaths from 10·01–12·00 years		2	1	4	5	6
	Deaths from melanoma	2 (13%)	1 (5%)	1 (4%)	1 (6%)	1 (6%)
	Deaths from other cause(s)	0	0	3 (16%)	4 (34%)	5 (32%)

Data at 2-year intervals from randomisation. Cutoff points for age were selected using quintiles rounded to the nearest whole number. Deaths presented as n or n (%).
