# Supplementary material for: Wide versus narrow excision margins for high-risk, primary cutaneous melanomas: long-term follow-up of survival in a randomised trial
Source: Lancet Oncol. 2016 Feb;17(2):184–92. doi: 10.1016/S1470-2045(15)00482-9 (PMC4737890; doi:10.1016/S1470-2045(15)00482-9)
Supplement: Supplementary appendix [file mmc1.pdf]

THE LANCET Oncology

Supplementary appendix

This appendix formed part of the original submission and has been peer reviewed. We post it as supplied by the authors.

Supplement to: Hayes AJ, Maynard L, Coombes G, et al, and the Scottish Cancer Therapy Network. Wide versus narrow excision margins for high-risk, primary cutaneous melanomas: long-term follow-up of survival in a randomised trial. *Lancet Oncol* 2016; published online Jan 11. http://dx.doi.org/10.1016/S1470-2045(15)00482-9.

Results from test of proportionality using Schoenfeld residuals

	Overall survival	Melanoma-specific survival
**Univariable analysis**	0.74	0.53
**Multivariable model**	0.29	0.13

Note: P-values from Chi^2 test

Results of Fine and Gray model for the subdistribution hazards

		Melanoma deaths				Non-melanoma deaths			
		HR	95% CI		P value	HR	95% CI		P value
**Margin**	**3cm**	1				1			
	**1cm**	1.25	1.00	1.58	0.055	0.85	0.59	1.23	0.39
**Sex**	**Female**	1				1			
	**Male**	1.33	1.03	1.71	0.027	1.07	0.73	1.56	0.74
**Tumour thickness* (mm)**		1.21	1.12	1.32	<0.0001	0.94	0.83	1.07	0.34
**Ulceration**	**Absent**	1				1			
	**Present**	1.71	1.35	2.15	<0.0001	1.05	0.73	1.53	0.78
**Site**	**Distal limb**	1				1			
	**Proximal limb**	1.49	1.07	2.08	0.019	0.86	0.53	1.38	0.52
	**Trunk**	1.74	1.29	2.34	<0.0001	0.70	0.45	1.08	0.11
**Age category**	**<60 years**					1			
	**≥60 years**	NA	NA	NA	NA	3.96	2.528	6.204	<0.0001

*Tumour thickness categorised as 0-2.49mm, 2.5-3.49mm, 3.5-4.49mm, 4.5-5.49mm, ≥5.5mm and fitted as linear effect

Sites recruiting patients

Hospital	PIs	N recruited
The Marie Sklodowska-Curie Memorial Cancer Center, Warsaw, Poland	W. Ruka, Z. Nowecki	108
Norfolk & Norwich University Hospitals, Norfolk, Norwich	T. O’Neill, A. Bardsley, A. Logan, M. Meyer	86
Derriford Hospital, Plymouth, Devon	J. Evans, D. Hanley, D. Harris	75
University Hospital, Sunderland, County Durham	R. B. Berry, M. Erdmann, J. James, J. Langtry, G. Rao	54
Mount Vernon, Northwood, Middlesex	B. Morgan, P. Cousins, D. Gault, A. Grobellaar, D .Harrison, D. Ross, P. Sanders, P. Smith	46
Scottish Melanoma Group	U. Chetty, M. Davies, J. Holmes, A. M. Morris, A. Nassan, J. H. Stevenson, A. D. Wilmhurst	44
Radcliffe Infirmary, Oxford	D. Coleman, T. Goodacre, H. Giele, S. Wall	39
Royal Victoria Infirmary, Newcastle upon Tyne	M. Black, P. Hodgkinson, C. Lawrence, M. Dahl, R. Milner, S. Pape	39
Frenchay Hospital, Bristol	J. Kenealey, N. Mercer, P. Townsend, D. A. Burd	34
Whiston Hospital, Prescot, Merseyside	R. Curley, R. Green, K. Graham, J. Bryson, K. Hancock, M. James	33
Nottingham University Hospital (Queens Medical Centre), Nottingham	A. Perks, J. Daly	29
Addenbrooke’s Hospital, Cambridge	P. Hall, B. Lamberty, G. Cormack	29
Royal Devon & Exeter, Exeter, Devon	J. Palmer, V. Deveraj	29
Royal Marsden Hospital, London	J. M. Thomas	20
Westminster & Chelsea, London	J. M. Thomas	20
Brighton General Hospital, Brighton, Sussex	C. Darley, M. Price, P. Hale	19
Glan Clwyd District General Hospital, Rhyl, Wales	C. Davies	15
Southampton University Hospitals, Southampton, Hampshire	J. Smallwood	14
The Ulster Hospital, Dundonald, Belfast	M. Brennan, D. Gordon, A. Leonard, A. Small	13
Salisbury District Hospital, Salisbury, Wiltshire	R. McDowall, M. Cadier, J. Hobby, D. McNeill, L. Rossi	12
Stoke Mandeville Hospital, Aylesbury, Buckinghamshire	A. Heywood, P. Budny	12
Frimley Park Hospital, Frimley, Surrey	R. Lallemand, I. Laidlaw	11
St George’s, Tooting, London	B. Powell	9
Wrexham Maelor Hospital, Wrexham	K. Crumplin, J. Sowden, J. Pye, P. Richards, M. Rosenberg	9
Kingston Hospital, Kingston on Thames, Surrey	C. Cahill, R. Leach	8
Kent and Sussex Hospital, Tunbridge Wells, Kent	A. Cook	7
Leicester Royal Infirmary, Leicester	H. Henderson, D. Ward	7
Royal Berskshire Hospital, Reading, Berkshire	M. James, D. Goodwin	6
The Royal London Hospital, London	J. Newton-Bishop	5
Coleraine Hospital, Londonderry, N Eire	F. Mullan	4
Royal Bournemouth Hospital, Dorset	S. Parvin	4

St Luke's Hospital, Bradford, Yorkshire	M. Timmons	4
King's College Hospital, London	A. du Vivier	4
Royal Naval Hospital, Haslar, Hampshire	R. Ashton	4
Royal Surrey Hospital, Guildford, Surrey	M. Kissin	4
St Mary's Hospital, London	D. Rosin	4
Walsgrave Hospital, Walsgrave, Coventry	A. Ilchyshyn	3
Sandwell District General Hospital, Lyndon, West Bromwich	J. Porter	3
North Hampshire Hospital, Basingstoke, Hampshire	A. Stebbing, H. Fawcett	3
Charing Cross Hospital, London	D. Davies	3
Singleton Hospital, Swansea, Wales	D. Roberts	3
Ipswich Hospital, Ipswich, West Suffolk	A. Bardsley, T. Cutler	2
St James's University Hospital, Leeds, Yorkshire	J. Newton-Bishop	2
Morriston Hospital, Swansea, Wales	M. Cooper, C. Gibbons	2
Groote Schuur, South Africa	C. Johnson	2
Middlesbrough General Hospital, Middlesbrough, South Tees	K. Sarang, C. Ramanathan	2
Queen Mary's Hospital, London	B. Powell, J. Clarke	2
Colchester General Hospital, Essex	B.. Sommerlad	2
St Thomas's Hospital, London	D. Ross	1
Crawley Hospital, Crawley, Sussex	A. Ball	1
Kingston General Hospital, Hull	N. Hart	1
Lister Hospital, Stevenage, Hertfordshire	M. Dickson	1
Horton Hospital, Banbury, Oxfordshire	M. Orr	1
St John's Hospital, Edinburgh, Scotland	Y. Wilson	1
Pinderfields Hospital, Wakefield, Yorkshire	L. Fourie	1
Royal Preston Hospital, Preston, Lancashire	J. Laitung	1
Ysbyty Gwynedd, Bangor, Wales, Bangor, Wales	A. MacFarlane	1
St. Andrew's Hospital, Billericay, Essex	M. Gittos	1
Thanet Hospital, Margate, Kent	P. Pheils	1
